# A facile method for the detection of DNA by using gold nanoparticle probes coupled with dynamic light scattering

**DOI:** 10.1186/1556-276X-7-564

**Published:** 2012-10-10

**Authors:** Yang Zhang, Wei-Wei Fei, Neng-Qin Jia

**Affiliations:** 1 The Education Ministry Key Laboratory of Resource Chemistry, Department of Chemistry, College of Life and Environmental Sciences, Shanghai Normal University, 100 Guilin Road, Shanghai, 200234, China

**Keywords:** DNA detection, Gold nanoclusters, Dynamic light scattering

## Abstract

In this paper, we present a simple and rapid method for deoxyribonucleic acid (DNA) detection using gold nanoparticle probes coupled with dynamic light scattering (DLS) analysis. The redox agent 1,4-dithio-dl-threitol cross-links the gold nanoparticles (AuNPs) to form clusters, while the monothiol DNA could terminate the formation and stabilize the assembled clusters by their negatively charge-based repulsions. By varying the concentration of DNA, the different sizes of DNA-AuNP clusters can be obtained. The sizes of the DNA-AuNP clusters were determined by DLS. A linear correlation was obtained between the sizes and the logarithm of DNA concentration from 2 nM to 5 μM with a detection limit of 1 nM (*S*/*N* = 3).

## Background

Gold nanoparticles (AuNPs), including spherical particles, nanorods, and nanoshells with sizes ranging from 10s to 100s nm, have attracted enormous attention in the recent years due to their unique properties, such as the quantum size effect, remarkably enhanced surface-to-volume ratio, and surface plasmon resonance [1-3].

The light-scattering cross section of gold nanoparticles (AuNPs) with a diameter of 60 nm is 4 to 5 orders of magnitude stronger than that of a strong fluorescence dye, e.g., fluorescein [4]. Plasmonic nanoparticles, such as AuNPs and silver nanoparticles, can be used for colorimetric detection, and its color change can be easily observed with the naked eye [5,6]. This results in a color change providing a simple, sensitive colorimetric method useful for many applications, such as metal cation [7,8], small molecule [9,10], protein [11,12], and cell imaging [13].

Dynamic light scattering (DLS), known as photon correlation spectroscopy, is a well-established noninvasive technique for measuring the size of particles in the range from 0.5 nm to 6 μm [14-18]. This technique is based on the Brownian motion of spherical particles which causes a Doppler shift of incident laser light. The diffusion constant of particles is measured, and the size of the particles is calculated according to the Stokes-Einstein relation [19]. DLS is a powerful tool for determining small changes in the size of particles. This technique couples the use of AuNPs probes as a light-scattering enhancer and DLS as a read-out system. The subsequent average particle size increase was then measured by DLS and correlated to the analyte concentration. Therefore, it is a potential analytical tool for quantitative immunoassay.

In this paper, we reported a facile and convenient method for the detection of deoxyribonucleic acid (DNA) based on the aggregation of AuNPs from a well-dispersed state. DNA functionalized AuNPs have been often used as colorimetric probes for DNA detection [20]. DLS is a sensitive method to determine small changes in the size of AuNPs, and the size distribution of Au nanoclusters has a linear response to DNA concentration in a wide range. Therefore, DLS can be a very sensitive technique for quantitative detection of DNA, and it opens many more possibilities in biomolecular analysis.

## Methods

### Materials

HAuCl_4_·4H_2_O and 1,4-dithio-dl-threitol (DTT) were obtained from Sinopharm Chemical Reagent Co., Ltd. (Shanghai, China). Tri-sodium citrate (C_6_H_5_Na_3_O_7_·2H_2_O) was purchased from Shanghai Chemical Reagent Company (Shanghai, China). Single-stranded monothiol DNA (TAACAATAATCCCTC-SH) was obtained from Shanghai Sangon Biological Engineering Technology & Services Co., Ltd (Songjiang, Shanghai, China). All other chemical reagents were of analytical grade, and all the solutions were prepared with double-distilled water.

### Apparatus and measurements

Transmission electron microscopy (TEM) was taken with a JEOL model JEM2100 (JEOL Ltd., Tokyo, Japan) transmission electron microscopy and operating at 200 kV. Field emission scanning electron microscopy (FESEM) image was performed using a JSM-840 field emission SEM system (JEOL Ltd., Tokyo, Japan). Ultraviolet (UV)-Vis absorption spectra were recorded by Thermo Multiskan spectrum (Thermo Fisher Scientific, Tai To-ku, Tokyo, Japan). Dynamic light scattering and surface potential were obtained from Malvern Zetasizer Nano ZS 90 (Malvern Instruments Ltd., Malvern, Worcestershire, UK).

### Preparation of AuNPs

AuNPs were prepared by the citrate reduction of HAuCl_4_[21]. Briefly, a solution of tri-sodium citrate (1 mL, 1% (*w/v*)) was rapidly added to a vigorously stirred boiling aqueous solution of HAuCl_4_ (50 mL, 0.01% (*w/v*)), which resulted in a change in solution color from deep red to purple. The solution was kept boiling for an additional 15 min. Then the solution was cooled to room temperature and filtered, the AuNPs were obtained and stored at 4°C for further use.

### Preparation of DNA-AuNP clusters

The DNA-AuNP clusters were prepared according to the literatures [22,23]. Briefly, 50 μL of the DNA solution with different concentrations was combined with 4 μL of aqueous DTT solution (800 μM) and added to 1 mL of AuNPs solution. This mixture of solution was incubated at 37°C for 90 min and then the unconjugated free DNA was removed by the centrifugation. The different sizes of DNA-AuNP clusters were obtained and stored at 4°C for further use.

According to the literature [22], when DTT and DNA were added to an AuNPs solution, the dithiol molecule, DTT, cross-links the AuNPs to form clusters; while the monothiol DNA was immobilized onto the AuNPs, which could terminate the formation and stabilize the assembled clusters by their negatively charge-based repulsions. The schematic illustration of the synthesis of DNA-AuNP cluster conjugates was shown in our previously reported literature [23]. Therefore, by fixing the concentration of DTT and varying the concentration of DNA, the different sizes of DNA-AuNP clusters can be obtained. The size of the DNA-AuNP clusters was determined by DLS.

## Results and discussion

### Characterization of AuNPs

**Figure 1:**
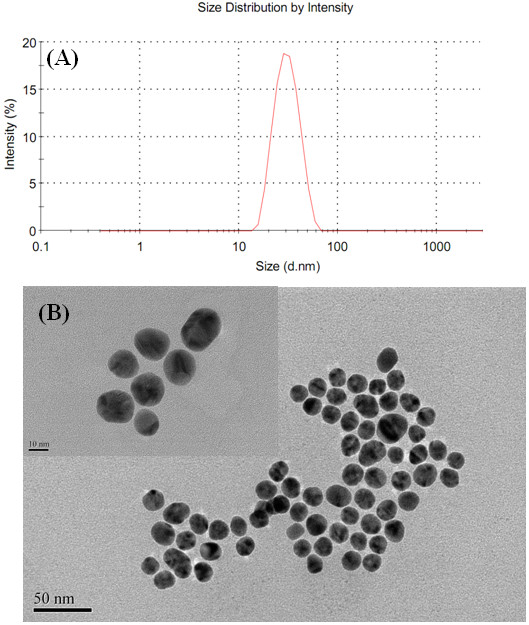
**Sizes of the prepared AuNPs.** (**A**) The DLS size distribution and (**B**) TEM images of the AuNPs.

The size of the prepared AuNPs was determined by DLS and TEM. As can be seen from Figure 1A, the hydrodynamic diameter of the AuNPs was estimated to be approximately 27 nm, while the TEM image of the AuNPs was shown in Figure 1B, which agrees well with the DLS analysis results.

### UV-visible absorption spectra and colorimetric detection of DNA-AuNP clusters

**Figure 2:**
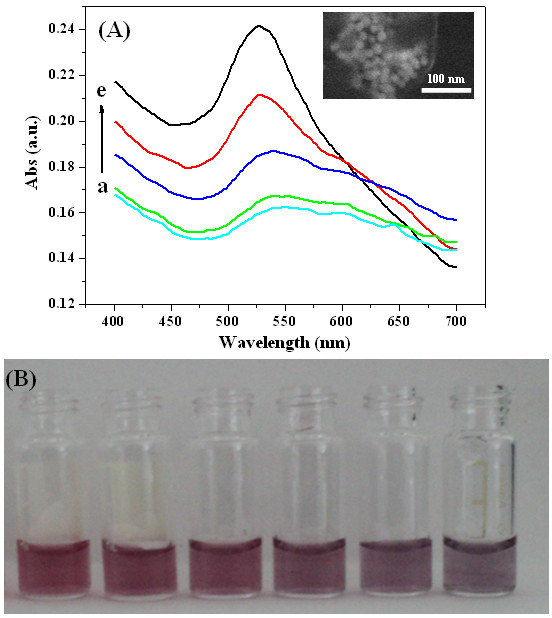
**UV-Vis absorption spectra of DNA-AuNP cluster conjugates with different colors.** (**A**) UV-Vis absorption spectra of DNA-AuNP cluster conjugates. The concentration of DTT is 800 μM and the concentration of DNA (from a to e) are as follows: 2 nM, 10 nM, 100 nM, 1 μM, and 5 μM. Inset: FESEM image of DNA-AuNP clusters formed by the addition of 5 μM of DNA. (**B**) DNA-AuNP cluster conjugates with different colors. The concentration of DTT is 800 μM, the concentration of DNA from left to right is 10 μM, 5μM, 1μM, 100 nM, 10 nM, and 2 nM.

UV-Vis absorption spectroscopy has been used as a common tool to monitor AuNPs aggregation, because the aggregation causes a shift or broadening of the surface plasmon resonance of the AuNPs [24]. The AuNPs with 27 nm in diameter have an intense plasmon absorption at approximately 520 nm (data not shown). As can be seen from Figure 2A, by increasing the concentrations of DNA (from a to e) at a constant DTT concentration, the absorption band at 524 nm gradually increased and meanwhile, the shoulder absorption band at 600 nm gradually decreased. The absorption coefficients at 524 and 600 nm are related to the quantities of dispersed and aggregated gold nanoparticles, respectively. The change in plasmon absorption is attributable to electronic dipole-dipole interaction and coupling between plasmons of neighboring particles in the aggregates. The corresponding color change is shown in Figure 2B; as the concentration of DNA increased, the color of the mixed solution changed from purple gradually to red. The color varied sensitively, which offered a possibility for visual observation. Meanwhile, representative FESEM image (inset of Figure 2A) also clearly showed the formation of the aggregates based on DNA-AuNP clusters conjugates, which agrees well with the UV-visible absorption spectra and the following DLS analysis results.

### Detection of DNA by dynamic light scattering

**Figure 3:**
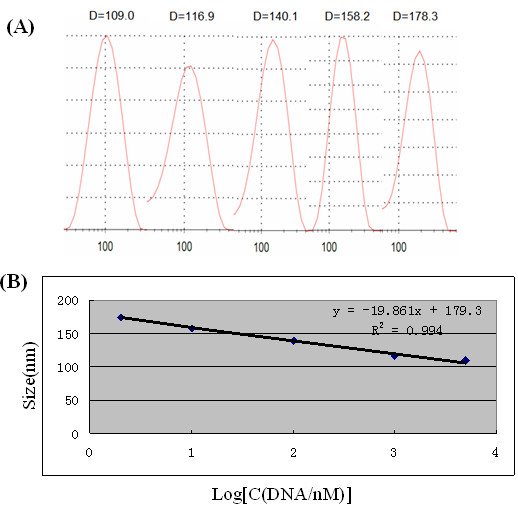
**Size distribution of the DNA-AuNP cluster conjugates, its linear relationship and logarithm of DNA concentration.** (**A**) The size distribution of the DNA-AuNP cluster conjugates in aqueous media was obtained by DLS. The concentration of DTT is 800 μM; from left to right, the concentration of DNA is 5 μM, 1 μM, 100 nM, 10 nM, and 2 nM. (**B**) The linear relationship between the DNA-AuNP cluster conjugates with different average diameters and the logarithm of DNA concentration.

DLS measurement was used to monitor the size change of AuNPs after conjugating with DNA. As shown in Figure 3A, the AuNPs assembled to form aggregates by adding DTT; the average hydrodynamic diameter of nanoparticle decreased as the concentration of DNA increased. The diameter of the DNA-AuNP clusters was determined to be 178.3 nm when the concentration of DNA was 2 nM, while the diameter was determined to be 109 nm when the concentration of DNA increased to 5 μM. Due to the repulsions induced by the charge of DNA, it can effectively stabilize the formed clusters and prevent them against larger aggregate formation.

As can be seen from Figure 3B, a linear correlation was obtained between the sizes and the logarithm of DNA concentration from 2 nM to 5 μM (*R*^2^ = 0.994), and a detection limit of 1 nM was obtained using the method of 3σ. The obtained results showed that DLS measurement could be used for DNA detection.

## Conclusions

In conclusion, by taking advantage of the large scattering cross section of AuNPs and the high sensitivity of DLS measurement, we demonstrated that DLS can be used very conveniently to quantitative studies of DNA. The size distribution of Au nanoclusters has a linear response to DNA concentration in a wide range. Our experimental results reported here offer a good platform for rapid, easy, and reliable detection of DNA. Therefore, DLS measurement opens up a new possibility for further study of other biomolecules.

## Competing interests

The authors declare that they have no competing interests.

## Authors’ contributions

YZ drafted the manuscript. WWF carried out the biological studies. NQJ conceived the study and participated in its design and coordination and helped draft and revise the manuscript. All authors read and approved the final manuscript.
